# Myofibroblasts: Function, Formation, and Scope of Molecular Therapies for Skin Fibrosis

**DOI:** 10.3390/biom11081095

**Published:** 2021-07-23

**Authors:** Yifan Tai, Emma L. Woods, Jordanna Dally, Deling Kong, Robert Steadman, Ryan Moseley, Adam C. Midgley

**Affiliations:** 1 Key Laboratory of Bioactive Materials for the Ministry of Education, College of Life Sciences, Nankai University, Tianjin 300071, China; 18342781135@163.com (Y.T.); kongdeling@nankai.edu.cn (D.K.); 2 Welsh Kidney Research Unit, Division of Infection and Immunity, Cardiff Institute of Tissue Engineering and Repair (CITER), School of Medicine, Cardiff University, Heath Park, Cardiff CF14 4XN, UK; WoodsE1@cardiff.ac.uk (E.L.W.); DallyJ2@cardiff.ac.uk (J.D.); SteadmanR@cardiff.ac.uk (R.S.); 3 Regenerative Biology Group, Oral and Biomedical Sciences, Cardiff Institute of Tissue Engineering and Repair (CITER), School of Dentistry, Cardiff University, Heath Park, Cardiff CF14 4XY, UK

**Keywords:** myofibroblast, fibrosis, wound healing, anti-scarring therapy, transforming growth factor-β1

## Abstract

Myofibroblasts are contractile, α-smooth muscle actin-positive cells with multiple roles in pathophysiological processes. Myofibroblasts mediate wound contractions, but their persistent presence in tissues is central to driving fibrosis, making them attractive cell targets for the development of therapeutic treatments. However, due to shared cellular markers with several other phenotypes, the specific targeting of myofibroblasts has long presented a scientific and clinical challenge. In recent years, myofibroblasts have drawn much attention among scientific research communities from multiple disciplines and specialisations. As further research uncovers the characterisations of myofibroblast formation, function, and regulation, the realisation of novel interventional routes for myofibroblasts within pathologies has emerged. The research community is approaching the means to finally target these cells, to prevent fibrosis, accelerate scarless wound healing, and attenuate associated disease-processes in clinical settings. This comprehensive review article describes the myofibroblast cell phenotype, their origins, and their diverse physiological and pathological functionality. Special attention has been given to mechanisms and molecular pathways governing myofibroblast differentiation, and updates in molecular interventions.

## 1. Myofibroblasts

In 1971, Gabbiani et al. identified large fibroblast-like cells within granulation tissue that had 40–80 A° diameter filamentous fibres traversing their entire cytoplasm. Since similar features are typical of smooth-muscle cells, the term ‘myofibroblast’ was coined (essentially, muscle-fibroblast intermediate cells) and it was proposed that these cells were implicated in wound contraction [1,2]. Further characterisation identified that the filamentous fibres were actin-based, with incorporated myosin and α-smooth muscle actin (α-SMA) proteins. Therefore, the myofibroblast’s ability to exert contractile force and to contract the wound edge was explained [3,4,5]. Myofibroblasts are morphologically enlarged and irregular (star or web-shaped) fusiform cells with well-developed cell–matrix focal interactions and intracellular gap junctions [6,7]. The incorporation of α-SMA into actin stress fibres grants the myofibroblast contractile power, approximately 2-fold that of the force of fibroblasts, when cultured on substrates with high elastomer stiffness [8,9,10]. Increased production of extracellular matrix (ECM) components: type I and type III fibrillar collagens, hyaluronan (HA), fibronectin (FN), and extra domain A fibronectin (EDA–FN) distinguish the hallmarks of myofibroblasts [11]. This elevated ECM content is not always causally linked, driving the myofibroblast differentiation process. Rather, there is a prominent interplay between ECM composition/arrangement and myofibroblast formation/function [12,13]. Following the delineation of the roles of myofibroblasts in wound contracture, they were quickly established as key drivers of progressive organ fibrosis [14], and have since been implicated in tumour development and metastasis [15,16]. More recently, studies describing an array of functions and regulatory factors exhibited by myofibroblasts have suggested roles beyond wound contraction and scar formation, including macrophage-like phagocytosis [17], immunomodulation [18,19], and autophagy [20]. In this review, we focus on the best described roles of myofibroblasts during wound healing and fibrosis. In addition, we summarise recent advancements towards targeting these cells with therapeutic molecular interventions.

## 2. Myofibroblast Origins

Multiple cell types are suggested to give rise to myofibroblasts, seemingly dependent on the tissue type. The heterogenous origins of myofibroblasts implies that these cells can form inside almost every tissue within the human body. In addition to resident fibroblasts and pericytes, their cellular origins include circulating bone-marrow-derived fibrocytes, tissue-derived mesenchymal stem cells, local epithelial and endothelial cells, de-differentiated smooth muscle cells, other hepatic stellate cells, mesangial cells, Schwann cells, and even monocytes and macrophages [21,22,23,24].

Differentiation from resident fibroblasts, present within most organs and connective tissues, is the best described process of myofibroblast development. Resting (inactivated) fibroblasts produce ECM and matrix proteases required for homeostatic turnover [25]. Upon activation, fibroblasts become highly migratory, proliferative, and increase the production of ECM, enzymes, and cytokines [26,27]; this transitional, activated state is termed the proto-myofibroblast. Despite limited information available in the literature regarding this phenotype, key features of the activated fibroblast, or proto-myofibroblast, have been described [28]. The rearrangement of the actin cytoskeleton from largely membrane-associated monomeric G-actin to polymerised cytoplasmic F-actin stress filaments, which traverse the length of the widened cell, is a hallmark feature [10]. These stress fibres allow junction formation with ECM components and other cells via integrin-containing complexes at the cell membrane and cadherin-type adherens junctions, respectively [7,29]. The absence of α-SMA within these stress fibres allows for proto-myofibroblast distinction from myofibroblasts [28]. Maturation into myofibroblasts can be determined by the neo-expression of α-SMA-positive stress fibres [30].

## 3. Myofibroblasts in Skin Fibrosis

The fibrotic process is characterised by chronic inflammation; altered epithelial–mesenchymal interactions; fibroblast proliferation, and fibroblast–myofibroblast differentiation. The latter feature (differentiation of fibroblasts into myofibroblasts) is central to the dysregulated and excessive production of collagen-rich ECM, otherwise called scar tissue. Myofibroblasts are thought to be terminally differentiated cells that typically undergo apoptosis [31,32,33,34] after wound contraction, as they are rarely found in non-pathological situations. Upon tissue trauma, myofibroblasts contribute to excessive ECM production for rapid, albeit dysfunctional, tissue repair. The tissue defect is repaired, but at the cost of function, as the organised spatial arrangement of specialised cells and ECM constituents is replaced by disorganised, abundant fibrous ECM. In this regard, the myofibroblast could be considered the primordial emergency repair cell. The aberrant persistence and chronic activation of myofibroblasts can lead to the development of pathological healing called fibrosis, which afflicts most tissues within the body. Progressive fibrosis leading to organ failure is considered the end-stage pathology of multiple diseases affecting the major organs, including but not limited to myocardial fibrosis [35], pulmonary fibrosis [36], liver cirrhosis [37], and chronic kidney disease [38]. Skin fibrosis (cicatrix) is an umbrella term for a large, heterogeneous spectrum of pathological conditions that affect the skin. Examples of skin conditions in which myofibroblast activity is central include hypertrophic and keloid scars, scleroderma, Dupuytren’s contracture, eosinophilic fasciitis, and chronic graft-versus-host disease [39,40,41,42,43,44]. The shared features of the abnormal and excessive accumulation of ECM constituents, particularly collagens, HA, and FN, in these diseases are governed by the myofibroblast phenotype. Infiltration of immune cells into fibrotic tissue also plays a key role in amplifying the fibrotic response, by secreting several cytokines and chemokines responsible for fibroblast–myofibroblast differentiation, the stimulation of ECM deposition, and the further recruitment of immune cells [45].

## 4. Mechanisms of Myofibroblast Formation

**Figure 1:**
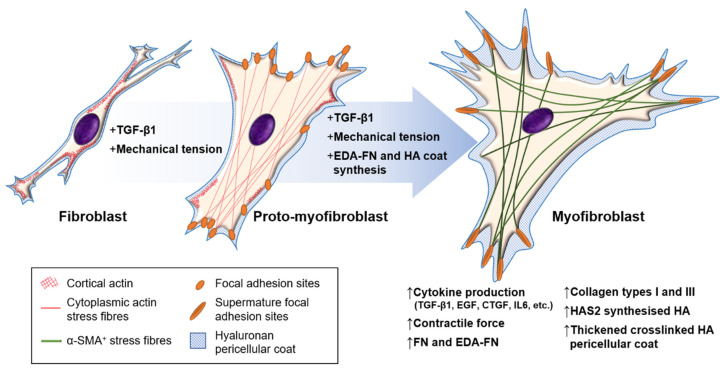
Fibroblast–myofibroblast differentiation. The process of fibroblast–myofibroblast differentiation requires: (i) the activation of the TGF-β1/Smad signalling pathway; (ii) cell–ECM mechanotransduction signalling; and (iii) the synthesis of modulators that promote and maintain the myofibroblast phenotype, such as EDA–FN and HAS2-synthesised HA. The intermediate stage is known as the proto-myofibroblast, and can be distinguished by increased proliferation, migration, and the rearrangement of cortical, membrane-associated actin into cytoplasmic filamentous actin stress fibres, which form focal adhesion sites at membrane–ECM junctions. The mature myofibroblast exhibits thick and crosslinked HA pericellular coats, and contains α-SMA^+^ cytoplasmic actin stress fibres that form mature focal adhesion plaques at membrane–ECM junctions and grant increased contractile strength.

At least three local events are needed to generate mature α-SMA-positive, fully differentiated myofibroblasts: (i) biologically active TGF-β1; (ii) extracellular stress, arising from the mechanical properties of the ECM (particularly collagen) and EDA–FN/integrin interactions; and (iii) the precursory production of phenotypic modulators (EDA–FN, HA) following activation (Figure 1). Increasing evidence strongly supports the role of inflammatory cell interactions in promoting myofibroblast development. Regardless of the origin, the resultant myofibroblast cells share the same properties and signalling cascade events that led to their formation.

### 4.1. The Canonical Pathway: TGF-β1/Smad

**Figure 2:**
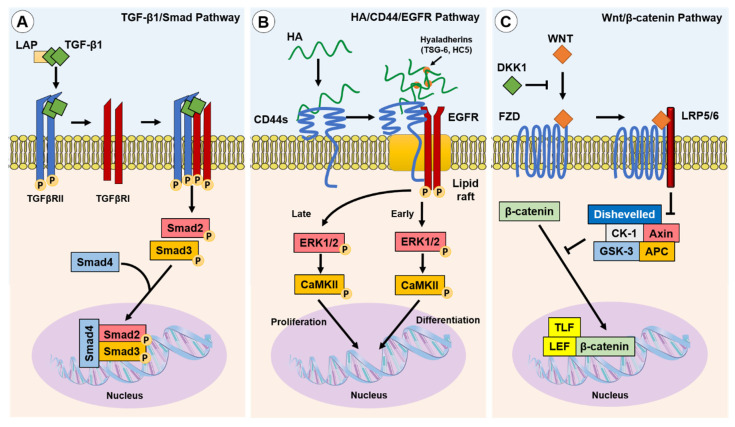
Canonical and non-canonical pathways implicated in fibroblast–myofibroblast differentiation. Three of the best described pathways involved in myofibroblast phenotypic acquisition are the TGF-β1/Smad; HA/CD44/EGFR; and Wnt/β-catenin pathways: (**A**) TGF-β1 is released from the LAP complex and binds to TGFβRII dimers, enabling association with and the activation of TGFβRI. Smad2 and Smad3 are subsequently phosphorylated and co-associate with Smad4 to initiate the transcription of pro-fibrotic genes; (**B**) TGF-β1 induced the upregulation of HAS2 synthesised linear HA and hyaladherin crosslinkers, such as TSG-6 and HC5, resulting in HA interactions with CD44s and the thickening of a crosslinked HA pericellular coat. HA interactions with CD44s induce clustering of CD44s within lipid rafts, wherein the co-receptor EGFR is activated and initiates biphasic signalling via ERK1/2 and CaMKII. Early signalling was suggested to promote differentiation-associated gene expression, whereas late signalling was suggested to promote proliferation-associated gene expression; and (**C**) the Wnt ligand binding to the transmembrane Frazzled receptor (FZD), in the absence of DKK1, which induces localisation with and the activation of LRP5 and LRP6. The Wnt/FZD/LRP signalling drives the dishevelled-mediated attenuation of the β-catenin inhibitory complex (CK-1/Axin/APC/GSK-3), facilitating the accumulation of β-catenin and its translocation to the nucleus, wherein it associates with co-transcription factors, TLF and LEF, to induce pro-fibrotic gene expression.

In classical cutaneous wound healing, tissue injury leads to TGF-β1 release from keratinocytes, macrophages and degranulating platelets [46]. In turn, TGF-β1 binds to and induces TGF-β receptor (TGFβR) I and II association on the fibroblast cell membrane, enabling TGFβRII phosphorylation of the TGFβRI kinase domain. In-turn, TGFβRI phosphorylates Smad2 and Smad3, which subsequently oligomerize with Smad4, forming trimeric protein complexes. These complexes are translocated to the nucleus, where they act as the transcription or co-transcription factors in the induction or repression of gene expression [47] (Figure 2A). Ultimately, TGF-β1/Smad pathway activation in fibroblasts leads to their differentiation into myofibroblasts [48]. TGF-β1 can also induce Smad-independent and co-receptor signalling pathways, such as mitogen-activated protein kinase (MAPK), p42/p44 extracellular signal regulated kinase (ERK1/2), Rho/Rho-associated protein kinase (ROCK), phosphatidylinositol-3-kinase (PI3K)/AKT, protein phosphatase 2A (PP2A), p38/c-Jun N-terminal kinase (JNK), protein kinase C (PKC), and tumour necrosis factor receptor associated factor (TRAF)-4/6 [49,50,51,52,53]. The tightly controlled TGF-β1/Smad-driven signalling events have been extensively researched within the context of myofibroblast differentiation and fibrosis, as highlighted in an eloquent review by Frangogiannis [54].

### 4.2. The Non-Canonical Pathway: HA/CD44/EGFR

A principal mediator of a non-canonical differentiation pathway in human myofibroblasts is HA, a glycosaminoglycan (GAG) formed of repeating d-glucuronic acid and N-acetyl-glucosamine disaccharide units. HA is synthesised by three HA-synthase (HAS) isoenzymes (HAS1, HAS2, HAS3) [55], and is metabolised by hyaluronidase (HYAL) enzymes (HYAL1, HYAL2, HYAL4) [56]. HA accumulation occurs simultaneously with upregulated HYAL1 and HYAL2 expression, but both these HA degrading enzymes have diminished expression in myofibroblasts. A subsequent decrease in HA degradation may also favour the accumulation of HA in fibrotic disease [57]. HAS2-synthesised HA is widely considered to be an important mediator of fibroblast–myofibroblast differentiation and EMT processes [58,59,60,61,62]. HAS2-synthesised linear HA forms a thickened pericellular coat that surrounds the activated fibroblast. This crosslinked and thickened HA pericellular coat, or glycocalyx, has been shown to be an essential structural element for human fibroblast–myofibroblast differentiation and phenotypic maintenance [59,63]. The HA pericellular coat remains thin in fibroblast phenotypes resistant to TGF-β1-driven differentiation, such as oral mucosal fibroblasts [64] and aged/senescent fibroblasts [58]. Interestingly, an enriched pericellular HA microenvironment is synthesised by foetal fibroblasts during foetal wound healing.

The HA pericellular coat is anchored to the fibroblast cell surface by the CD44 receptor [65,66], and crosslinked by hyaladherins [59]. Association with CD44 and other hyaladherins orchestrates the HA pericellular coat and results in the activation of downstream extracellular–intracellular signal cascades, shown to be prominent in disease pathogenesis including fibrosis, cancers, and inflammatory diseases [67,68]. CD44 exists as multiple alternatively spliced variants and all CD44 variants (CD44v), as well as the standard form (CD44s), possess HA binding motifs within their extracellular head domain. CD44v have variability within alternatively spliced exons of the extracellular stem region [69,70]. Studies have shown that silencing CD44 using small interfering RNA (siRNA) prevented the downstream intracellular signalling required for human fibroblast–myofibroblast differentiation and inhibited HA pericellular coat formation [71,72]. These studies concluded that HA/CD44 association was essential for human myofibroblast differentiation, with recent research showing that CD44s expressed on the surface of human fibroblasts was the dominant player in regulating HA coat-mediated myofibroblast differentiation [73]. HA-crosslinking hyaladherins include inter-α-inhibitor (IαI) heavy chains (HC), which are localised by association with the tumour necrosis factor-stimulated gene (TSG)-6 [74,75]. The increased presence of HA-associated HC and TSG-6 complexes have been associated with the pathology of multiple inflammatory diseases including arthritis, asthma, and some cancers [76]. Martin et al. identified that IαI-HC5 was covalently bound to chondroitin sulphate on bikunin and was essential for the formation of HA pericellular coats in myofibroblasts. Increased expression of TSG-6 following TGF-β1 activation of fibroblasts facilitated the transfer of HC5 to HA by TSG-6 catalysis, in a metal ion-dependent manner [77].

HA binding to CD44 plays a pivotal role in the intracellular signalling required for human fibroblast–myofibroblast differentiation [58,63,72]. CD44 was shown to be present throughout the membranes of fibroblasts but clustered into localised populations within myofibroblast membranes. These distinct areas were identified to be cholesterol-rich lipid rafts, and ‘locked’ clusters of CD44 within lipid rafts were shown to be dependent on HA binding and crosslinking into pericellular coats [72]. HA coat removal released CD44 from lipid rafts and re-enabled their movement through the membrane, but also inhibited downstream signalling cascades [63]. Further investigation revealed that CD44 was co-localised with epidermal growth factor (EGFR) within membrane lipid raft domains, which resulted in EGFR activation, and the phosphorylation of downstream effectors and transcription factors, ERK1/2 and calcium calmodulin kinase (CaMK)-II (CaMKII). The chemical inhibition of ERK1/2 or CaMKII prevented myofibroblast differentiation, and only when TGF-βRII and CD44/EGFR signalling were active could myofibroblast differentiation occur [72]. Both ERK1/2 and CaMKII were phosphorylated in a biphasic manner, with early and late-phase activation. Early activation of ERK1/2 (<5 min) was thought to be associated with eventual fibroblast–myofibroblast differentiation, whereas later activation (>30 min) was suggested to play a role mediating cellular proliferation responses [72] (Figure 2B). However, signalling functions appear to be cell-dependent as early, but not late, ERK1/2 signalling peaks were observed in TGF-β1-stimulated non-scarring oral mucosal fibroblasts; these cells exhibited an anti-proliferative response in a HA/CD44-independent manner [65].

### 4.3. The Wnt/β-Catenin Pathway

The Wnt signalling pathway is well described in its vital roles during embryogenesis and determination of cell fate, differentiation, proliferation, and apoptosis. We refer interested readers to a thorough review article on Wnt signalling functions [78]. Carefully orchestrated Wnt signalling has been found to be essential for tissue homeostasis, whilst the dysregulation of the Wnt pathway can result in pathogenesis [79,80]. Wnt signals simultaneously through the co-association of Frazzled receptors and low-density lipid protein co-receptors (LRP). The fate of β-catenin, an important regulator of Wnt signalling, is regulated by Dickkopf-related protein-1 (DKK1). DKK1 inhibits Wnt signalling by activating downstream signalling complexes, and results in β-catenin degradation. When DKK1 is not present, Wnt signalling leads to β-catenin translocation to the nucleus, its association with co-transcription factors, and the subsequent activation of Wnt-associated pro-fibrotic genes [78,79,81] (Figure 2C). The activation of Wnt signalling was implicated in the fibrogenesis of multiple organs [82]. Wnt-1 and Wnt-10b were noted to be overexpressed, whereas DKK1 was decreased, which led to the increased nuclear accumulation of β-catenin observed in human tissue samples from systemic scleroderma (SSc), idiopathic pulmonary fibrosis (IPF), and liver cirrhosis [82]. Wnt signalling stimulated by TGF-β1-induced inhibition of DKK1 was shown to induce myofibroblast differentiation, up-regulate the release of ECM components (notably collagens) and induce fibrosis [82,83,84]. In addition, crosstalk between the TGF-β/Smad pathway and the Wnt/β-catenin pathway is suggested to be a prominent mechanism in the development of hypertrophic and keloid scars [85].

### 4.4. Mechanotransduction

Mechanotransduction is the ability of stress force to convert extracellular to intracellular signalling. Fibroblasts can perceive external forces (mechanoperception) through their fibronexus structures in vivo or mature focal adhesion (FA) structures in vitro [86,87]. ECM rigidity determines the size of the cell’s FAs, or ‘anchors’, which in turn limit the level of tension generated within intracellular stress fibres. Only when substrate stiffness permits the formation of mature FAs (8–30 μm), and the generation of approximately four-fold greater stress compared with usual FAs (2–6 μm), does α-SMA become incorporated into pre-existing cytoplasmic β-actin stress fibres [88]. Thus, the myofibroblast phenotype is mechanosensitive. The transition of fibroblasts to the proto-myofibroblast state was suggested to be related to increased microenvironment stiffness [28,88]. TGF-β1 is locked within the ECM by latency-associated peptide (LAP) and latent TGF-β1-binding protein (LTBP), and is released by proteolysis or integrin-dependent mechanotransduction [89]; resulting in a feedback loop of pro-fibrotic fibroblast activity and increased ECM stiffness [29,90,91,92]. In a recent study, the balance of ECM composition, elasticity, and TGF-β1 signalling was shown to govern fibroblast phenotypic heterogeneity and give rise to distinct, but overlapping, fibroblast subsets [93]. Indeed, Kollmannsberger and colleagues showed that within 3D microtissues grown in vitro, fibroblasts transitioned to proliferative myofibroblasts at the growth front, where a high degree of tensile force and stretched FN fibres were present. As the tissue matrix matured into collagen-rich ECM with low FN fibre tension, more fibroblasts were present, suggesting that the myofibroblast phenotype stabilised by tensile forces could revert to a fibroblast phenotype by low tensile force [94]. These studies suggest that substrate stiffness is a key determinant of fibroblast and myofibroblast plasticity, and that reducing excessive mechanotransduction may be an option in controlling myofibroblast presence.

**Figure 3:**
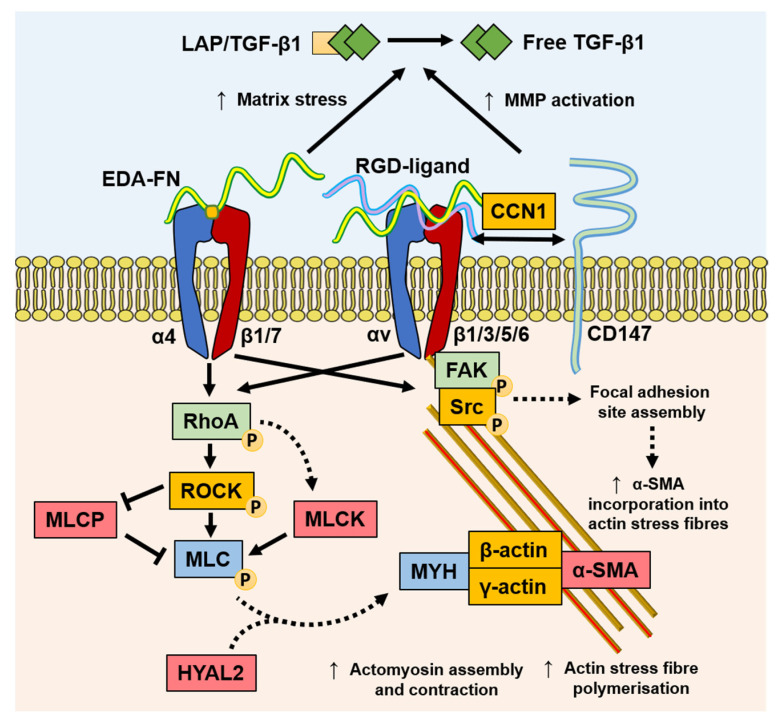
Mechanisms of mechanotransduction implicated in myofibroblast differentiation and function. TGF-β1 stimulates an increased expression of EDA–FN. The EDA segment contains the EDGIHEL motif, which binds to the integrin α4β1/α4β7 receptor cleft, initiating signalling through RhoA/MLCK and FAK. RhoA signalling results in myosin light chain (MLC) promotion of myosin (MYH)/actin assembly and stress fibre polymerisation, which is augmented by HYAL2 that relocates from the membrane to the cytoplasm. RGD motif containing ligands, such as FN and collagens, bind integrins and facilitate the formation of focal adhesion sites, increasing contractile force and α-SMA incorporation into actin stress fibres. In addition, the increased matrix stress generated by EDA–FN and increased MMP activation from CD147 recruitment exacerbates active TGF-β1 release from the extracellular LAP complex.

Myofibroblasts generate force through stress fibre contraction, and this is transmitted to the ECM via FAs containing transmembrane receptors, typically integrins [87,95], as summarised in Figure 3. Integrins mediate cell–cell, and especially, cell–ECM interactions, and are prominently involved in the initiation, maintenance, and resolution of fibrosis. EDA–FN is synthesised by fibroblasts and various other cell types [96,97]. The EDA–FN splice variant can only be detected during tissue repair [98,99], fibrosis [100], tumour development [101,102,103], and transiently during embryogenesis [104]. The accumulation of EDA–FN has been observed in multiple fibrotic disorders, including lung, liver, and skin [100,105,106,107]. EDA–FN promotes myofibroblast differentiation by orchestrating LAP/LTBP release and activation of TGF-β1 [108], increasing matrix stress–strain tension [109,110], and by activating mechanotransducer-integrin signalling via FAK activation [111,112,113,114]. Research by Shinde et al. [115] and Kohan et al. [112] demonstrated that fibroblast-expressed integrins, α4β1 (VLA-4) and α4β7 (LPAM-1), mediate different roles in myofibroblast formation, respectively. Integrin α4β1/EDA–FN promoted ECM synthesis and stiffening [115], whereas integrin α4β7/EDA–FN induced myofibroblast stress fibre formation and contractility [112]. The combined exclusivity of EDA–FN expression and the prominent roles it plays in driving the myofibroblast phenotype have highlighted the protein as an attractive target for anti-fibrotic interventional therapies.

Discoidin domain receptor (DDR)1 is a mediator of stromal–epithelial interactions, and belongs to the family of collagen receptors [116] overexpressed in pro-fibrotic keloid fibroblasts [117]. DDR1 mediates collagen contraction and stiffness, thereby indirectly mediating pro-fibrogenic responses that are largely independent from collagen binding to integrins [118]. Additionally, the spatial and structural properties of the local 3D collagen microarchitecture can distinctly affect fibroblast and myofibroblast activity. Collagen fibril alignment and diameter were shown to affect fibroblast contractility and migration via alteration in integrin clustering and the stability of adhesion sites [119]. Seo and colleagues suggested that collagen fibre thickness held more pertinence over dictating myofibroblast differentiation independently from collagen quantity [120]. Furthermore, collagen-rich ECM stiffening can also be potentiated by the myofibroblast and inflammatory cell production of collagen crosslinking enzymes, such as lysyl oxidases [11,90]. CD147, also known as extracellular matrix metalloproteinase inducer (EMMPRIN), is a well-established matrix metalloproteinase (MMP)-inducer that is rapidly becoming understood to mediate multiple cellular responses. The linked association of CD147 with integrin α6β1 was observed during increased metastasis in human hepatoma cells [121], suggesting a role for CD147 in modulating integrin α6β1 associations, with matricellular protein CCN1, and mechano-sensitivity to senescence or apoptosis [122]. Interestingly, the nullification of the extracellular domain of CD147 reversed associations with integrin β subunits and FA sites, suggesting that the extracellular domain of CD147 binds to integrins to regulate the mechanical tension of the ECM [121,123]. The roles of these mechanotransducer proteins in stress relay, which is involved in myofibroblast differentiation, will become clearer with continued investigation.

Increasing evidence suggests that myofibroblasts have the capacity for classical CaMK-myosin light chain kinase (MLCK)-dependent smooth muscle cell contraction mechanisms [124] and contractile activation by the RhoA/ROCK/myosin light chain phosphatase (MLCP) pathway [28,125,126], highlighting a key difference between the smooth muscle cell and myofibroblast contractile mechanisms. It was recently discovered that HYAL2, previously thought to only possess HA catalytic activity, was shown to have non-enzymatic functions [69,127,128]. Following TGF-β1 activation, enhanced HAS2 expression by fibroblasts leads to increased HA accumulation within the ECM observed in 3D matrices in vitro [129] and in fibrosis progression in vivo [130]. However, HYAL2 was shown to re-localize to the cytoplasm and align along the actin cytoskeleton in myofibroblasts [128], which may contribute to increasing extracellular HA accumulation. The cytoskeleton-aligned HYAL2 was associated with α-SMA, RhoA, and MLCK. The silencing of HYAL2 did not prevent but only delayed myofibroblast differentiation. The presence of HYAL2 was shown to accelerate RhoA/MLCK phosphorylation and cellular contractility, suggesting a role in the mechanotransduction and the orchestration of key cytoskeletal and FA-related proteins [128]. These early findings suggest that there may be complex orchestration roles that HYAL2 plays in cytoskeletal reassembly.

Other mediators of mechanotransduction and promising targets in mediating fibroblast–myofibroblast differentiation include myocardin-related transcription factor (MRTF) [131,132], Yes-associated protein (YAP) [133,134,135,136,137], cadherins [138], and Notch [139]. In the case of these multifunctional proteins, further exploratory research will help establish the feasibility of targeting their activity to interfere with myofibroblast differentiation or function.

## 5. Interventional Strategies to Target Myofibroblasts for Scarless Skin Healing

There are currently no clinically approved anti-scarring therapies specifically designed to limit or prevent dermal fibrosis. Several wound management strategies completed phase II trials, but did not advance to phase III trials in recent years. The clinical studies database, https://clinicaltrials.gov (‘Fibrosis, Skin’ searched 25 June 2021), showed 10 registered clinical trials that have either completed phase III or are currently in progress (Table 1). The proposed therapies include a range of treatment modes, including antibody and drug therapies, scar resection or reduction, and tissue-engineering strategies.

Rituximab is a mouse/human chimeric antibody that binds CD20 on the surface of B-lymphocytes and initiates cell apoptosis, complement activation, and cell-mediated cytotoxicity. B-cell depletion by rituximab was shown to reduce fibrosis serum markers and myofibroblast activation in IgG4-related inflammatory diseases [140]. The results from the phase II/III trial are yet to be released; however, rituximab was shown to reduce B-cell presence in autoimmune scleroderma skin tissues, but had no discernible benefits to skin scar tissue histology after 6 months of treatment [141], which suggests that rituximab may be more effective as a pre-emptive measure. The anti-fibrotic effect of kynurenic acid was also proposed to occur via the induction of apoptosis in leukocytes and a reduction in inflammation, but with the additional regulation of the fibroblast production of MMPs and collagens [142]. Pirfenidone has received extensive attention for its efficacious action in the treatment of IPF [143,144]. Recently, Chen et al. used in situ self-assembling HA lattice nanostructure spray-on dressings loaded with pirfenidone to treat deep partial-thickness burn injuries in mouse models. The addition of pirfenidone to the wound dressing reduced collagen accumulation and myofibroblast presence within wound sites [145]. Multiple ongoing clinical trials focus on the management of established scars, with the aim to reduce scar appearance or to induce physiological healing after scar resection. The combinatory treatment of radiation-induced fibrosis with pentoxifylline (a hemorheological agent) and vitamin E was suggested to exhibit synergistic improvement in scar appearance and ECM remodelling, through actions of increased tissue perfusion and free radical scavenging [146]. Carbon dioxide (CO_2_) ablative fractional laser therapy has emerged as a promising low-invasiveness therapy for the induction of collagen remodelling, resulting in scar revision and reduced hypertrophic scar appearance after multiple sessions [147]. Integra is a biosynthetic dermal substitute wound dressing widely used in acute wound management; the ongoing clinical trial aims to perform scar resection and uses Integra to encourage attenuated scar healing, in a similar manner to previously shown outcomes of Integra-treated post-burn scar resections [148]. Autologous skin cells were harvested at the point of graft surgery, using ReCell technology [149]. The cells were applied to chronic wounds prior to the grafting of autologous donor skin, which resulted in remarkable healing results [150]. The ongoing clinical trial will attempt to recapitulate this improved healing response following scar resection in patients with hypertrophic scars.

There are numerous records of clinical trials aimed at one or more of the multifaceted aspects of skin fibrosis. However, progression to phase III trials appears to be rarely achieved, perhaps owing to the limited experimental evidence of myofibroblast-specific targets. Below, we highlight progresses in the experimental and pre-clinical evaluation of candidate molecular-based therapies that target key myofibroblast functions or activities, to prevent skin fibrosis.

### 5.1. Experimental and Pre-Clinical Interventions for Myofibroblast Differentiation

Popularised pre-clinical strategies for preventing myofibroblast formation focus on inhibition of the canonical TGF-β1/Smad signalling pathway using cytokines, antibodies, and regulators of gene expression. Demonstrating the clinical efficacy of anti-TGF-β1 therapies has been hindered by the cytokine’s pleiotropism and ubiquitous presence, as well as the heterogenous processes of healing and fibrosis. Consequently, therapeutic targeting of TGF-β and its specific isoforms has proved challenging. Available clinical data are limited to early phase trials and studies with conflicting outcomes [54]. Recombinant human TGF-β3 (Avotermin, Juvista) was developed by Renovo and hailed as a potential novel inhibitor of dermal scar formation, based on its elevated presence and roles in non-scarring tissues compared to TGF-β1, such as early gestational foetal skin and the oral mucosa [151,152,153,154]. Phase I/II clinical trials concluded that the intradermal application of Juvista yielded both short- and long-term improvements in scar appearance, compared to placebo and standard wound care management [155,156]. Unfortunately, Juvista did not meet the necessary primary or secondary endpoints required by phase III trials, failing to demonstrate reduced scarring after excisional surgery. Fresolimumab is a neutralising antibody that targets all TGF-β isoforms; the effects of Fresolimumab on cutaneous SSc revealed reduced expression of fibrotic biomarkers and diminished myofibroblasts [157]. This suggested that a shotgun approach to TGF-β inhibition had anti-fibrotic effects, whilst neutralising TGF-β1 alone in SSc failed to demonstrate clinical efficacy [158]. An apparent trend has emerged, wherein anti-scarring interventions that target an individual aspect or factor of fibrotic conditions generally fail to realise clinical benefit. Moreover, the efficacious index of growth factors is limited by their low stability, short half-life in vivo, adverse effects arising from elevated local and/or systemic concentrations, and their non-specific nature (multiple protein-binding partners) [159].

MicroRNAs (miRNA) are short non-coding RNAs (18–25 nucleotides) that bind the 3′ untranslated region of mRNA, thereby inhibiting mRNA translation or promoting mRNA degradation. Multiple studies have shown the regulatory roles of miRNA in pathological wound healing and skin fibrosis. Notable miRNA targets upregulated in skin fibrosis include miRNA (miR)-21 [160] and miR-130b [161], whereas miR-29 [162], miR-129-5p [163], and miR-7 [164,165] were shown to be negative regulators of myofibroblast activity and fibrosis. The miRNAs, miR-21 and miR-17-5p, have been shown to be directly implicated in TGF-β1/Smad pathway promotion. Smad2 activity was potentiated by these miRNAs through their inhibition of Smad7 [166,167]. Thus, inhibiting the activity of these miRNAs has arisen as a potential avenue in gene therapies to mitigate TGF-β1/Smad pathway activity and myofibroblast differentiation. We refer readers to an elegantly written review of miRNAs in various fibrotic diseases for a more in-depth discussion of the miRNAs involved in modulating TGF-β1 signalling [168].

Bone morphogenetic protein-7 (BMP7) exerted potent anti-fibrotic actions in vitro [169], and in pre-clinical fibrosis models [170,171,172,173]. BMP7 activation of Smad1/5/8 results in the competitive binding of Smad4, thereby inhibiting the TGF-β1/Smad2/3 pathway’s transcriptional activities [173]. Early reports indicated that BMP7 failed to prevent bleomycin-induced skin fibrosis [174]. However, a later study showed that BMP7 treatment at the point of thermal injury prevented hypertrophic scar formation [173]. Thus, BMP7 therapies require further optimization and testing to determine their effectiveness in the prevention of dermal fibrosis. Interferon (IFN)-γ induced the expression of Smad7, which antagonised interactions between Smad2/3 and TGFβRI/II [175]. The effects of IFN-γ on fibroblasts in vitro are conflicting; exogenous IFN-γ was suggested to abrogate TGF-β1-induced proliferation, migration, and differentiation to myofibroblasts [176]; but T-cell secretion of IFN-γ, found to be prominent in SSc, aggravated fibrosis by promoting fibroblast proliferation and collagen synthesis [177]. Early pre-clinical and small clinical studies suggested that IFN-γ was effective in the attenuation of lung, renal, and liver fibrosis [178,179,180], and oral submucosal fibrosis [178,179,180,181]. There is a current lack of studies describing definitive IFN-γ mechanisms involved in the regulation of myofibroblasts. Thus, whether IFN-γ delivery would be effective in attenuating dermal fibrosis remains unclear.

Alternative approaches include molecular inhibitors of non-canonical and mechanotransduction pathways to regulate ECM composition and disrupt the myofibroblast phenotype. Hepatocyte growth factor (HGF) was first implicated in liver regeneration [182], but has since been shown to prevent fibrosis initiation and progression in animal models [183,184]. HGF treatment was shown to attenuate collagen production by fibroblasts in multiple tissues [185,186,187]. Elevated HGF expression in fibrosis [188,189,190,191], but also in differentiation-resistant fibroblasts [192,193], suggests a duality of HGF actions which may be a consequence of truncated isoforms possessing a variable number of kringle domains (HGF/NK1-4) with differential signalling activities. Indeed, human oral mucosal fibroblast resistance to TGF-β1-induced differentiation was dependent on heightened HGF and HGF/NK1 expression, whereas HGF/NK2 was preferentially expressed by dermal fibroblasts [194]. Recently, HGF/NK1 gene therapy was shown to exert potent anti-fibrotic effects through attenuated collagen types I, III, and IV deposition in mouse models of renal fibrosis [171]. In addition, the HGF inhibition of collagen synthesis and promotion of MMP production has shown promise for therapeutic applications in reducing the pro-fibrotic activity of keloid fibroblasts in vitro and in a keloid heterograft mouse model [195,196,197]. Small molecule inhibitors of DDR1 have shown promise in reducing collagen types I and IV deposition in bleomycin-induced renal and lung fibrosis [198,199,200]. Ongoing research into DDR1-specific inhibitors have yielded promising results in models of renal fibrosis [199].

HA bioactivity is dependent on its molecular weight, enzymatic synthesis, and endogenous versus exogenous application [55,201,202]. The disruption or prevention of HA pericellular coat synthesis using hyaluronidase enzymes [203], HAS inhibitors [204,205] or exogenous HA oligosaccharides [59] results in the failure of TGF-β1-stimulated myofibroblast phenotypic acquisition. The inhibition of HAS2 activity [206] or global HAS synthesis of HA [204,205] have demonstrated preventative effects in various models of fibrosis. However, given the ubiquitous role of HAS-synthesised HA in tissues, and the lack of specific HAS isoenzyme inhibitors, it is unclear at present whether the inhibition of HA synthesis would be beneficial in a clinical setting. An additional action of BMP7 involved the induction of the nuclear translocation of HYAL2 and the subsequent splicing of CD44 mRNA, which resulted in the upregulated expression of the variant isoform, CD44v7/8. Fibroblasts with upregulated expression of CD44v7/8 exhibited ‘HA-phage’ activity, wherein the HA pericellular coat was rapidly internalised and broken down, resulting in the destabilization of the myofibroblast and phenotypic reversion to fibroblasts [69,169]. Whether CD44v7/8-dependent actions convey anti-fibrotic effects in dermal fibrosis is currently unknown.

The most promising integrin targets that have demonstrated roles in the pathogenesis of fibrosis are α4-containing integrins (α4β1/7) [112,115] and αv-containing integrins (αvβ1/3/5/6) [207,208,209]. Mechanotransduction by integrin αvβ6 promoted traction from proliferating liver cholangiocytes to FN/LAP, which subsequently released active TGF-β1 and initiated the differentiation of surrounding hepatic stellate cells to myofibroblasts [207,210]. A small peptidomimetic, EMD527040, mimics the RGD-binding sites of αvβ6 and αvβ1; orally administered EMD527040 attenuated biliary and non-biliary fibrogenesis [207]. The expression of integrin αvβ6 was elevated in keratinocytes during wound healing and fibrosis [211], but the detailed mechanistic roles of integrin αvβ6 and epithelial–mesenchymal crosstalk during dermal fibrosis have yet to be elucidated. Research has identified that integrin interactions with the EDGIHEL motif of EDA–FN was causative of downstream profibrotic responses [109,113,115]. The IST-9 [109,110], F8 [212,213], and vaccine-generated [214] antibodies that target EDA–FN or integrin α4 have demonstrated prevention of fibroblast–myofibroblast differentiation [109,110]. Therapeutic applications of antibodies can be limited by the cost and complexity of production, off-target immune activation or unspecific protein masking of critical protein–protein interactions. To address these issues, Zhang et al. developed a small blocking polypeptide to bind and block EDA–FN interactions with the α4β1 binding cleft, with high specificity [114]. The polypeptide, AF38Pep, was designed to mimic the integrin α4β1 receptor site for the EDGIHEL motif of EDA–FN and was shown to interfere with TGF-β1-stimulated fibroblast–myofibroblast formation by the specific blockade of integrin α4β1 signalling, the inhibition of FAK activation, and the prevention of profibrotic gene transcription [114]. The aforementioned first generation of small blocking polypeptides have revealed the renewed promise of the specific interruption of integrin-mediated mechanotransduction and myofibroblast formation. The implementation of integrin receptor peptidomimetics for the prevention of skin fibrosis will become more apparent in the coming years when the in vitro research progresses into pre-clinical models.

Another potential peptide therapy is the N-terminal amino acid sequence of α-SMA, Ac-EEED, which is important for the tropomyosin-1.6/7-stabilized incorporation of α-SMA into cytoplasmic stress fibres [215]. Interestingly, the cytoplasmic delivery of the Ac-EEED peptide resulted in the loss of α-SMA from β-cytoplasmic actin stress fibres and inhibited G-actin polymerization into F-actin [216,217], thereby reducing myofibroblast contraction in wound healing [218]. Ac-EEED peptide therapy has yet to be evaluated in skin fibrosis models, which may be related to the current lack of available dermal myofibroblast-specific targeting moieties that operate by endosomal uptake and the cytoplasmic release of payloads.

### 5.2. Immunomodulating Biomolecules for Fibrosis Attenuation

Immunoregulatory interventions aim to control the inflammatory phase in postnatal skin healing to attenuate scar formation [219,220]. Studies in transgenic mice provided early indicators that the absence of neutrophils and macrophages led to scar-free healing [221]. Targeted repression of the gap junction and inflammatory mediator protein, connexin-43, supported these findings [220,222]. Additionally, connexin-43 was shown to mediate cardiac fibroblast–myofibroblast differentiation [223] and promote aberrant cardiomyocyte–myofibroblast functional coupling [224]. Certain interleukin (IL) cytokines are implicated in the activation of inflammatory cascades. IL-8 production is a chemoattractant to neutrophils, whereas IL-6 secretion by fibroblasts activates macrophages and monocyte chemotaxis. Both IL-6 and IL-8 exhibit the rapid induction of expression following tissue injury, resulting in the recruitment of circulating inflammatory cells. The expression levels of IL-6 and IL-8 are elevated and maintained for longer in adult skin, compared to scarless scar-free foetal repair. The inhibition of phosphodiesterase 4 (PDE4) reduced scar formation in skin fibrosis models by interfering with the release of IL-6 from M2 macrophages [225]. Thus, IL-6 and IL-8 are considered pro-fibrotic mediators, whereas IL-10 antagonised their activity [219,226]. IL-10 gene therapy resulted in reduced inflammation and the promotion of scarless healing in mouse wound healing studies [227]. The exogenous addition and macrophage paracrine production of IL-10 were shown to induce myofibroblast reversal to fibroblastic phenotypes in vitro [228,229]. More recently, IL-10 induced myofibroblast–fibroblast dedifferentiation was shown to alter dynamic interactions with the surrounding fibrillar matrix with a demonstratable loss of contractility in IL-10 treated myofibroblasts [230]. Research into inflammation-induced fibrosis has revealed additional potential candidate ILs that may be targetable in skin fibrosis, including IL-11 [35], IL-16 [231], and IL-33 [232]. Keratinocytes have suggested roles in the regulation of the myofibroblast phenotype and profibrotic ECM during wound healing [233,234,235]. More recently, the keratinocyte secretion of IL-1α was demonstrated to restrain the myofibroblast phenotype, dependent on fibroblast integrin α4β1 expression and Cox-2/Nrf2 signalling [236,237].

The importance and therapeutic potential of macrophages in the wound healing process has been highlighted in recent years [6,238,239]. Crosstalk between myofibroblasts and macrophages during skin repair was reported [240]. Growth factors secreted by CD301b^+^ M2-type macrophages were shown to selectively stimulate the proliferation of adipocyte precursor (AP)-derived myofibroblasts only. In aged mice wounds and experimentally induced mouse skin fibrosis, AP-derived myofibroblasts and CD301b^+^ macrophages were reduced, and a CD29^+^ myofibroblast pool was increased. In keloids, CD301b^+^ macrophages and AP-derived myofibroblasts were also increased [241]. In fibrotic lung tissues, cadherin-11 mediated the adhesion between macrophages and myofibroblasts, promoting pro-fibrotic myofibroblast activity via the paracrine release of TGF-β1 by the macrophages [242]. Experimental research has suggested roles of mast cells in scar formation [243]; reduced numbers of activated mast cells were reported to improve healing and minimize scarring [244,245,246], whereas mast cell hyperplasia was causally linked to myofibroblast hyperplasia [247,248,249]. Increased neuropeptide activity and the presence of substance-P (SP) were found in hypertrophic scar samples [250,251]. Mast cells were previously suggested to be a major source of neuropeptide SP-stimulated inflammation and increased myofibroblast activity [252]. These studies showed that myofibroblast activity could be mediated through regulated leukocyte–myofibroblast interactions and suggest that targeting certain leukocyte subpopulations may serve as anti-fibrotic strategies.

Targeting inflammatory meditators has shown promise in alleviating the magnitude of fibrosis in various animal models. Follistatin-like-1 (FSTL1) was found to be elevated in serum from patients with silicosis and in mouse lung fibrosis models. FSTL1-induced IL-1β production by macrophages and positively regulated TGF-β1 signalling in fibroblasts. The inhibition of FSTL1 expression or activity protected against lung injury and fibrosis [253,254]. Recently, FSTL1 neutralizing antibodies were shown to exhibit potent anti-inflammatory actions, attenuate bleomycin-induced IPF and dermal fibrosis in vivo, and downregulate TGF-β1-driven fibrosis in human skin ex vivo [255].

The transmembrane serine protease and collagenase, fibroblast activation protein (FAP) is prominently expressed by activated fibroblasts and myofibroblasts during tissue remodelling and fibrosis [256]. FAP-cleaved collagen binds to the scavenger receptor (SR)-A, recruiting SR-A^+^ macrophages to sites of collagen turnover [257]. The liver expression of FAP in cirrhosis was shown to correlate with the severity of fibrosis but was not exclusively expressed by α-SMA^+^ myofibroblasts, suggesting that FAP marks a differentially activated fibroblast state [258]. Lines of research have started to establish the mechanistic actions of FAP in fibroblast heterogeneity and governance over the pro-fibrotic ECM [93]. Treatment with anti-FAP antibody reduced collagen type I production by fibro-stenotic intestinal myofibroblasts [259]. The FAP and dipeptidyl peptidase IV inhibitor, talabostat mesylate (PT100), was used to treat bleomycin-induced IPF murine models. Treatment with PT100 showed anti-fibro-proliferative activity but increased macrophage activation, with no effect on collagen expression [260]. Therefore, the present scope for specific FAP inhibition in dermal fibrosis models remains unclear, until more mechanistic information is reported.

### 5.3. Targeted Myofibroblast Apoptosis

In physiological wound healing, myofibroblasts disappeared following wound closure and resolution [122,261], predominantly by apoptosis [33]. Despite the elevated production of reactive oxygen species (ROS) by myofibroblasts during fibrosis, the persistence of TGF-β1 expression, ECM deposition, and accumulative stress-induced FAK promotes pro-survival and anti-apoptotic myofibroblast phenotypes [31,262]. The susceptibility of myofibroblasts to nitric oxide (NO)-induced apoptosis has been reported in vitro [263]. Therefore, a combination of reduced profibrotic growth factor expression, increased ECM turnover, and increased NO generation may set the stage for triggering myofibroblast apoptosis during the resolution of tissue repair and remodelling [264,265].

A single chain antibody (C1-3) specifically targets synaptophysin^+^ liver myofibroblasts without co-localising with liver monocytes or macrophages [266], thus demonstrating that the identification of unique markers of myofibroblasts in fibrosis could serve as targeting devices. The researchers showed that C1-3-gliotoxin conjugates induced non-parenchymal cell apoptosis and depleted liver myofibroblasts without affecting monocytes or macrophages, resulting in the reduced severity of fibrosis [266]. The anticancer drug, Elesclomol, was found to selectively induce apoptosis in activated fibroblasts and myofibroblasts isolated from scar tissue samples. Elesclomol upregulated intracellular levels of ROS, caspase-3, and cytochrome-c proteins, resulting in reduced myofibroblast numbers and a lower scar elevation in vivo [267].

The mechanical tension-stimulated myofibroblast differentiation increased mitochondrial priming and death signalling proteins, such as the pro-apoptotic BH3-only protein BIM [268,269]. The anti-apoptotic protein BCL-X_L_ sequesters BIM and ensures myofibroblast survival. Lagares et al. showed that myofibroblasts were susceptible to apoptosis induced by the BCL-2 inhibitor and the BH3 mimetic drug, ABT-263 (Navitoclax), which inhibited BCL-X_L_ and allowed BIM to activate myofibroblast apoptosis in mouse models of scleroderma dermal fibrosis [32]. These results were recapitulated in rabbit ear hypertrophic scar models, wherein ABT-263 improved scar appearance and collagen arrangement [40]. Future studies into the physiological triggers for time-appropriate myofibroblast apoptosis could potentially lead to the identification of novel treatments with improved therapeutic indexes for scarless wound healing.

### 5.4. Antioxidant Therapeutics

Another proposed regulator of normal and pathological scarring in numerous tissues is oxidative stress, referring to the overproduction of ROS via such mechanisms as NADPH oxidases (NOXs) and the mitochondria, at the expense of cellular and tissue antioxidant defences [270,271,272,273,274]. The induction of the myofibroblast phenotype is accompanied by depleted cellular antioxidants, leading to ROS generation and the implication of ROS in multiple signalling pathways associated with myofibroblast differentiation. Thus, an emergent area of research is focusing on evaluating the efficacious index of antioxidants against fibrosis and in restoring cellular redox balance. The liposomal delivery of copper/zinc (Cu/Zn) superoxide dismutase (SOD)-attenuated TGF-β1, α-SMA and collagen type I expression in dermal fibroblasts, although myofibroblast apoptosis remained unaffected [275]. Similarly, SOD1-containing fusion proteins alleviated oxidative stress in cardiac myofibroblasts via the reduced expression of TGF-β1, α-SMA, and collagen types I and III—whilst restoring MMP-1 and attenuating MMP inhibitor (TIMP-1) secretion [276]. Small molecule inhibitors of NOXs, such as GKT136901 and GKT137831, also exhibited therapeutic potential by reducing murine liver fibrosis [277,278]. In addition to the progress made with such promising findings, the development of nanoparticles with inherent antioxidant activities, such as cerium oxide, fullerene, and mesoporous silica, have also been explored as potential therapeutic options for fibrosis, which may also be combined with payloads of pharmaceuticals, genes, or proteins [279].

Natural compounds have a long history of use in wound healing, and the cellular mechanisms of action are beginning to be delineated as active components are extracted and assessed [280,281,282]. The exploitation of the aromatic nature and antioxidant capabilities of various naturally sourced polyphenolic compounds and their extracts have been demonstrated in numerous in vitro and in vivo systems, with desirable biocompatible and anti-fibrotic effects in models of pulmonary [283,284,285]; renal [286,287]; myocardial [288,289]; and skin fibrosis [290,291]. The clinical potential of natural compound-based therapies is often restricted by the lack of clarity in their multifaceted bioactivities. Hence, delineating cellular responses to the more specific and potent actions of natural compound extracts has become a popularised concept towards their clinical translation.

## 6. Future Perspectives for the Discovery of Novel Therapeutics

The complex multistage process of wound healing is vulnerable to dysregulation by a plethora of factors that can initiate and maintain myofibroblast differentiation. Failure of timely wound resolution and myofibroblast apoptosis can inevitably result in the excessive and disorganised deposition of the collagen-rich ECM that is characteristic of fibrosis. The growing knowledge base surrounding myofibroblast cell formation, function, and profibrotic regulators of differentiation has revealed promising candidate therapies that target the myofibroblast phenotype and functions at various stages of wound healing, with the aim to achieve attenuated scar formation. In the wake of unsatisfactory pre-clinical and clinical trial results from the inhibition of TGF-β1, researchers have sought alternative routes to preventing myofibroblast differentiation. Fibroblasts resistant to TGF-β1-stimulated myofibroblast differentiation, such as embryonic fibroblasts, oral mucosal fibroblasts, and aged/senescent fibroblasts, which continue to be extensively studied to uncover important targets, including cytokines, ECM components, receptors, and intracellular signalling cascades involved in resistance to myofibroblast phenotypic acquisition. Fundamental research into molecular-based interventions have given rise to an abundance of candidate treatment modalities, but a key limitation remains: namely a deficit of cell-specific targeted therapies due to the current lack of identified markers unique to myofibroblasts, perhaps as a result of their heterogenous origins. Biomaterials and drug carrier technologies may offer circumvention by facilitating the localised release of bioactive molecules within the microenvironment and the vicinity of myofibroblasts and are likely to take precedence in the sophisticated and controlled spatiotemporal delivery of therapeutic agents to wound sites. The progress and increased accessibility of single cell analytics and multi-omics may help to further identify subsets of dermal fibroblasts (e.g., fibroblasts from papillary or reticular dermis) or myofibroblasts that have more prominent roles in driving fibrosis, and this may facilitate the discovery of uniquely expressed cell surface receptors, proteins, or other targetable moieties. An alternative strategy only briefly touched upon here includes the regulation of cell–cell dynamics. Research into direct heterogenous cell binding, cell paracrine activity, and influence on surrounding cells may provide further clues towards myofibroblast regulation (e.g., leukocyte, stem cell, keratinocyte/epithelial cell, or endothelial cell regulation of fibroblasts and vice versa). Regardless of the afflicted tissue, the underlying cellular aetiology of fibrosis is largely similar. This undoubtedly implies that the progress of research in organ fibrosis will also provide applicable therapies for scarless wound healing. Here, we provided a comprehensive overview of promising candidate molecular-based therapies, which have been directly inspired by the discovery of key regulatory mechanisms implicated in myofibroblast formation and function. Certainly, continued research into myofibroblast biology will usher in a new era of novel therapeutics that will, in-turn, contribute to the knowledge pool. This review highlights the current literature and understanding of cellular mechanisms and interventions of myofibroblasts in the context of reduced scar skin repair, and our hope is that it serves as a reference guide for ongoing and planned research.

## Figures and Tables

**Table 1:** Recent clinical trials for attenuated scar skin repair (completed phase III or ongoing).

Treatment (Mechanism)	Type of Skin Fibrosis	Phase Status	ClinicalTrials.gov ID
Rituximab/CD20 mAb (immunomodulation)	Scleroderma, SSc	Phase II/III (2019)	NCT04274257
FS2/kynurenic acid (immunomodulation)	Scar, hypertrophic scar, graft scar, keloid	Phase II (ongoing)	NCT04186273
Pirfenidone (unknown mechanism)	Scar	Phase II/III (ongoing)	NCT03068234
Pentoxifylline, vitamin E (ECM remodelling)	Postoperative radiation-induced fibrosis	Phase III (ongoing)	NCT02898376
Fractional CO_2_ laser (ECM remodelling)	Scar, burns, graft scars	N/A (ongoing)	NCT04456127
Integra skin substitute (scar resection and healing)	Scar	N/A (ongoing)	NCT04420442
Autologous cell dermal scaffolds (scar resection and healing)	Scar, hypertrophic scar	N/A (ongoing)	NCT04389164

## Data Availability

No new data were created or analysed in this study. Data sharing is not applicable to this article.
